# Effects of Arsenite Resistance on the Growth and Functional Gene Expression of *Leptospirillum ferriphilum* and *Acidithiobacillus thiooxidans* in Pure Culture and Coculture

**DOI:** 10.1155/2015/203197

**Published:** 2015-05-12

**Authors:** Huidan Jiang, Yili Liang, Huaqun Yin, Yunhua Xiao, Xue Guo, Ying Xu, Qi Hu, Hongwei Liu, Xueduan Liu

**Affiliations:** ^1^School of Minerals Processing and Bioengineering, Central South University, Changsha, Hunan 410083, China; ^2^Key Laboratory of Biometallurgy of the Ministry of Education, Changsha, Hunan 410083, China

## Abstract

The response of iron-oxidizing *Leptospirillum ferriphilum* YSK and sulfur-oxidizing *Acidithiobacillus thiooxidans* A01 to arsenite under pure culture and coculture was investigated based on biochemical characterization (concentration of iron ion and pH value) and related gene expression. *L. ferriphilum* YSK and *At. thiooxidans* A01 in pure culture could adapt up to 400 mM and 800 mM As(III) after domestication, respectively, although arsenite showed a negative effect on both strains. The coculture showed a stronger sulfur and ferrous ion oxidation activity when exposed to arsenite. In coculture, the pH value showed no significant difference when under 500 mM arsenite stress, and the cell number of *At. thiooxidans* was higher than that in pure culture benefiting from the interaction with *L. ferriphilum*. The expression profile showed that the arsenic efflux system in the coculture was more active than that in pure culture, indicating that there is a synergetic interaction between *At. thiooxidans* A01 and *L. ferriphilum* YSK. In addition, a model was proposed to illustrate the interaction between arsenite and the *ars* operon in *L. ferriphilum* YSK and *At. thiooxidans* A01. This study will facilitate the effective application of coculture in the bioleaching process by taking advantage of strain-strain communication and coordination.

## 1. Introduction

As well known, acidophilic microorganisms inhabit some of the most metal-rich environments. Although metal resistance systems have been studied in neutrophilic microorganisms, it is only in recent years that attention has been placed on metal resistance in acidophiles [1]. Arsenic (As) is a well-known environmental toxicant which can impair the physiology of most organisms [2], and its dissolution could inhibit bacterial activity in the bioleaching process. It is one of the most prevalent and highly toxic substances that often coexist with minerals in the natural environment [3]. Previous investigations have revealed that some metal ores contain variety of arsenic containing metal sulphides, such as arsenopyrite in gold ores. During mineral pretreatment, large quantities of arsenic are released into continuous-flow aeration tanks in which the biooxidation takes place [4]. The high toxicity of dissolved arsenic in the system can seriously inhibit the microbial activity and even completely stop the biooxidation process [5]. In order to improve leaching efficiency and recover metals from the leachates, strong resistance is necessary for bioleaching microorganisms [1]. The two soluble inorganic forms of arsenic are As(III) and As(V) in the bioleaching environment, with the former being more toxic [6, 7]. As(III) is reported to inhibit the growth of bacteria to a degree greater than As(V) [8]. Therefore, we investigated the As(III) resistance of acidophilic bacteria in pure culture and coculture in this study.

Several mechanisms to cope with arsenic compounds are known to operate in microorganisms. These mechanisms include extracellular precipitation, chelation and intracellular sequestration, and active extrusion from the cell or biochemical transformation (redox or methylation) [9]. Arsenic efflux systems are widespread in most sequenced bioleaching organisms. They were encoded by* ars* operon, containing three genes: a transcriptional regulator (*arsR*), a transmembrane pump (*arsB*), and an arsenate reductase (*arsC*) [1, 10]. ArsC reduces As(V) to As(III) prior to efflux via a membrane potential driven pump (ArsB) controlled by a transacting repressor (ArsR) [11, 12]. ArsC is the most important protein of arsenic detoxification [13]. ArsC and the primary and secondary carrier proteins are located in the plasma membrane function to extrude arsenite outside of the cell [14, 15]. The* arsB* gene encodes a membrane protein that can function independently as a chemiosmotic arsenite transporter [13, 15]. ArsR is a transacting regulatory protein which controls its own expression and an As(III)-responsive transcriptional repressor that binds to the* ars* promoter. Binding of arsenite to ArsR results in dissociation of the repressor from the DNA and hence gene expression [15, 16]. Kotzé revealed that* arsRC* gene of* Acidithiobacillus ferrooxidans* is closely related to the* ars* genes which were functional when transformed into an* Escherichia coli ars* deletion mutant ACSH50Iq and conferred increased levels of resistance to arsenate and arsenite [17]. The function of ArsH protein in the context of the arsenic resistance pathway was ambiguous [18], though recent studies placed it in the family of the NADPH-dependent FMN (flavin mononucleotide) reductases [19, 20]. Acidophilic arsenic resistance operons have been cloned from* A. caldus* and* L. ferriphilum* [4, 21]. There is no report on arsenic resistance of* At. thiooxidans* yet.

Single bioleaching of refractory sulfide ores was comprehensively studied in the past decades [22]. However, it encountered a lot of problems in practical applications. Metabolism process of single strain is simple and ineffective in the use of energy substances, so that environmental adaptability is relatively weak, which results in low leaching efficiency. Then, strain–strain communication and coordination in mixed culture gained considerable attention in recent years because of their abilities to send, receive, and to process information, increasing the chances of survival in complex environments [23]. It was reported that the mixed culture possessed good leaching effect, which was more efficient than the single one [24, 25]. The majorities of arsenopyrite biooxidation processes operate at 40°C and are dominated by a mixture of sulfur-oxidizing bacterium and iron-oxidizing bacterium. The moderately thermophilic bacteria, such as* L. ferriphilum* [26, 27] and* At. thiooxidans* [28], showed high copper recoveries, which draws attentions in this field. However, there has not been a correlation between the dominant bacterial species and the efficiency of the bioleaching process [24].

The aim of this investigation was to determine the response of iron-oxidizing* L. ferriphilum* YSK and sulfur-oxidizing* At. thiooxidans* A01 to arsenite in pure culture and coculture. The cell growth, ferrous iron, and sulfur oxidation of two acidophilic bacteria in pure and coculture were investigated. The expression profile of arsenic resistance genes in* L. ferriphilum* YSK and* At. thiooxidans* A01 upon arsenite shock was analyzed for the first time. In addition, a model was established to describe the possible arsenic detoxification pathway in two strains. Coculture system sheds the light on our understanding of interactions between acidophilic organisms under the stress of arsenite, which will facilitate its effective application in the bioleaching process by taking advantage of catabolic diversity.

## 2. Materials and Methods

### 2.1. Microorganisms

The bacteria used in the experiment were* Leptospirillum ferriphilum* (YSK, DQ981839) and* Acidithiobacillus thiooxidans* (A01, FJ154526), which were isolated by our laboratory from representative mineral resources in China.

### 2.2. Pure Culture and Coculture Conditions

The pure culture of* L. ferriphilum* YSK and* At. thiooxidans* A01 was grown in 9K basal salt medium containing 44.7 g/L FeSO_4_·7H_2_O and 10 g/L sulfur in a shaker incubator set at 30°C and 170 rpm, respectively. The 9K medium contained the following (g/L water): KCl, 0.1; (NH_4_)_2_SO_4_, 3.0; MgSO_4_·7H_2_O, 0.5; KH_2_PO_4_, 0.05; Ca(NO_3_)_2_·4H_2_O, 0.01; and its pH was adjusted to pH 2.0 with dilute H_2_SO_4_. The medium was autoclaved at 121°C for 20 min.

The growth rate of* L. ferriphilum* YSK and* At. thiooxidans* A01 is proportional to ferrous and sulfur oxidation, respectively. Coculture was grown in 9K medium containing both ferrous iron and sulfur. The effect of 44.7 g/L FeSO_4_·7H_2_O and 0–10 g/L sulfur on the growth of* At. thiooxidans* A01 and* L. ferriphilum* YSK was examined, respectively. The modified 9K medium, which is composed of 44.7 g/L FeSO_4_·7H_2_O and 5 g/L sulfur and serves as the energy source, was employed to improve coculture growth. In pure culture and coculture, the initial cell density of each strain after inoculation was 1.0 ± 0.5 × 10^7^ cells/mL.

### 2.3. Metal Tolerance Study

To study the influence of arsenite on bacterial growth and oxidative capacity, pure culture and coculture were exposed to 0, 100, 200, 300, 400, 500, 600, 700, 800, and 900 mM sodium arsenite, respectively.* L. ferriphilum* YSK and* At. thiooxidans* A01 were first cultivated separately in medium without arsenite until the middle logarithmic phase and then inoculated to 100 mL medium containing 100 mM As(III), respectively. The inoculation for coculture with 200 mM As(III) was from pure culture of* L. ferriphilum* YSK and* At. thiooxidans* A01 grown in 100 mM As(III). Similarly, this experimental setup was applied to study the influence of elevated sodium arsenite. All experiments were conducted in triplicate. After exposure to ultraviolet light for 30 min, NaAsO_2_, sublimed sulfur, and FeSO_4_·7H_2_O were added to the previously autoclaved 9K medium.

### 2.4. Arsenic-Shock Treatment

According to the results of metal tolerance experiment, pure culture and coculture entered early logarithmic growth phase 48 h after inoculation. Individual 100 mL bacterial culture in early-log-phase (48 hours) in 250-mL flask was shocked by 300 mM NaAsO_2_. A parallel identical experiment without NaAsO_2_ was performed as a control. Every experiment was conducted in triplicate. Once the treatment reached 10, 30, 60, and 120 minutes, cells were harvested for RNA extraction.

### 2.5. Cell Collection and DNA Extraction

Cells were collected from* L. ferriphilum* YSK and* At. thiooxidans* A01 in pure culture and coculture supplemented with 0 and 300 mM As(III). After going through a filter paper, the culture was centrifuged at 1,000 ×g for 15 min in Avanti J-E centrifuge (Beckman Coulter, USA). The precipitation was rinsed twice with diluted H_2_SO_4_ (pH = 2.0) and centrifuged at 1,000 ×g for 15 min [29]. Then, the combined supernatant was centrifuged at 15,000 ×g for 25 min. The resulting pellet from pure culture and coculture was used to extract total DNA, using TIANamp Genomic DNA purification kit (Tiangen Biotech, Co., Ltd., Beijing, China) according to the manufacturer's instructions. DNA samples were visualized on a 1% (w/v) agarose gel with ethidium bromide staining.

### 2.6. Total RNA Extraction, Purification, and cDNA Generation

Cells for RNA extraction were filtered to discard jarosite and sublimed sulfur and then centrifuged at 1,000 ×g for 15 min in Avanti J-E centrifuge (Beckman Coulter, USA). The Total RNA was extracted and purified according to the method described by Wang et al. [30]. About 1 *μ*g purified RNA from each sample was used for cDNA synthesis with the ReverTra Ace qPCR RT Kit (TOYOBO), according to the manufacturer's protocol. RNA extracts were treated with DNase to remove DNA before cDNA synthesis.

### 2.7. Primers and Real-Time PCR

The genome of* L. ferriphilum* YSK (GenBank accession number: CP007243) was fully sequenced and completely annotated. The draft genome sequence of* At. thiooxidans* A01 was deposited at DDBJ/EMBL/GenBank under the accession number AZMO00000000 [31]. Arsenic resistance-related genes in both strains were figured out based on the genome sequences. Specific primers of* L. ferriphilum* YSK and* At. thiooxidans* A01 used in this study were shown in Table 1. The specificity of primers was checked by conventional PCR and sequencing. Conventional PCR products amplified from genome DNA were purified by E.Z.N.A.TM gel extraction kit (Omega Bio-tek, USA), and a series of tenfold dilutions were made and used as the template to construct standard curves. Real-time PCR was performed using the MyiQ Real-Time PCR Detection System and analyzed using the MyiQ software package (Bio-Rad Laboratories, Inc.). Primer efficiencies were determined using the formula efficiency (*E*) = 10^(−1/slope)^, with the slope determined by the MyiQ Cycler software. The determination of the threshold values (Ct) was generated automatically by the MyiQ software [32]. Each 25 *μ*L of reaction mix was composed of 12.5 *μ*L 2× SYBR Green Real-time PCR Master Mix (Toyobo co., Ltd., Osaka, Japan), 2 *μ*L template cDNA, 0.5 *μ*L of each forward primer and reverse primer (the stock concentration is 10 *μ*M), and 9.5 *μ*L nuclease-free ddH_2_O. The PCR program started with an initial denaturation at 95°C for 5 min and 40 cycles of 95°C for 20 s, 57°C for 15 s, and 72°C for 30 s. The constitutively expressed* gyr*B gene was used as reference to determine the relative abundance of arsenic-related genes. In addition, quantitative PCR (qPCR) of* gyr*B gene was applied to measure the dynamics of pure culture and coculture.

### 2.8. Analytical Methods

The concentration of ferrous irons was measured by titration with potassium dichromate (K2Cr2O7) twice a day in a period of 10 days. The pH value was measured daily with a pH-meter (ZD-2A, REX Instrument Factory, Shanghai). Cell numbers were counted using a hemocytometer with a light microscope at 100x magnification at 24 h interval.

## 3. Results

### 3.1. Coculture Establishment

**Figure 1:**
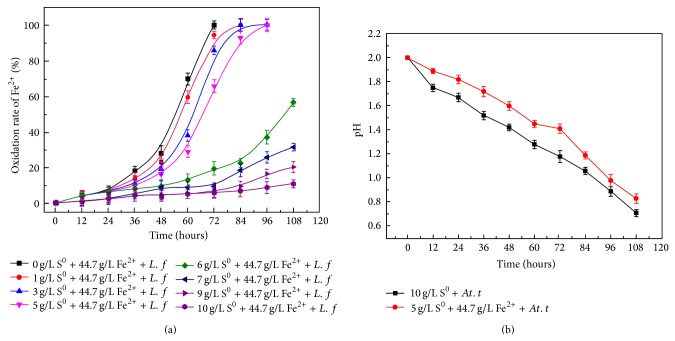
Ferrous iron oxidation rate of* L. ferriphilum* YSK in pure culture with sulfur (a) and pH value of* At. thiooxidans* A01 in pure culture containing ferrous sulfate (b). The error bars indicate the standard deviation in triplicate.

In order to construct a coculture system, ferrous iron oxidation of* L. ferriphilum* YSK exposed to sulfur and sulfur oxidation of* At. thiooxidans* A01 with ferrous iron was studied. According to the results (Figure 1(a)), ferrous sulfate oxidation of* L. ferriphilum* YSK without sulfur was 100% after 72 h. With the increase of sulfur, ferrous sulfate oxidation gradually slowed down. When 5 g/L sulfur was added, ferrous iron was totally oxidized 108 h after inoculation. Once sulfur ≧6 g/L, less than 60% ferrous iron was oxidized in 108 h, indicating the growth of* L. ferriphilum* YSK was significantly inhibited. Compared to 9k medium containing 10 g/L sulfur, 5 g/L sulfur and 44.7 g/L FeSO_4_·7H_2_O showed no significant impact on the growth of* At. thiooxidans* A01 (Figure 1(b)). So, coculture was successfully constructed with the modified 9K medium containing 44.7 g/L FeSO_4_·7H_2_O and 5 g/L sulfur as energy sources.

### 3.2. Physiology Characteristics of Pure Culture and Coculture in Response to As(III) Stress

The ferrous iron oxidation and pH change of* L. ferriphilum* YSK and* At. thiooxidans* A01 in pure culture and coculture upon arsenite stress were investigated.

**Figure 2:**
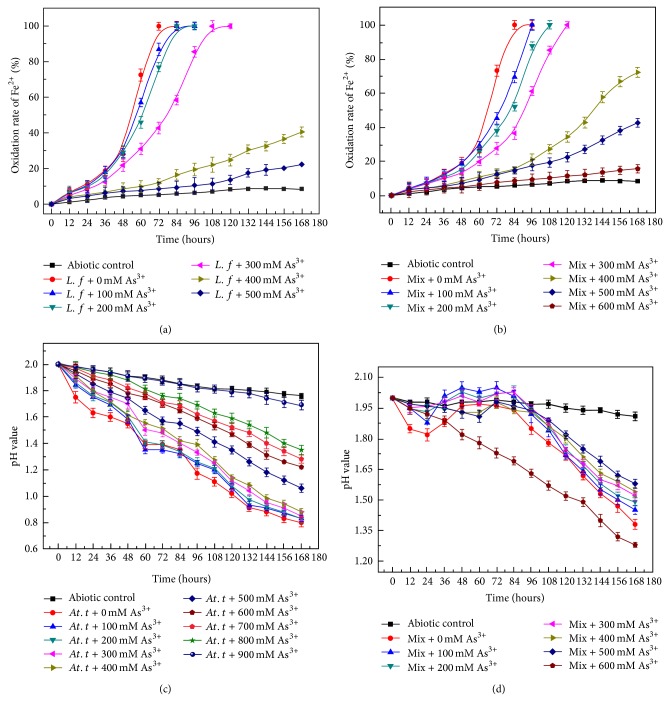
Ferrous iron oxidation rate of* L. ferriphilum* YSK in pure culture (a) and coculture (b) upon As(III) stress; pH value of* At. thiooxidans* A01 in pure culture (c) and coculture (d) upon As(III) stress. The error bars indicate the standard deviation in triplicate.

With the increase of arsenite ion, ferrous oxidation rate of* L. ferriphilum* YSK in pure culture and coculture gradually slowed down (Figures 2(a)-2(b)). Ferrous iron was totally oxidized within 72 h in pure culture and 84 h in coculture without As(III). The oxidation rate of ferrous in pure culture (from 12 to 72 h) and coculture (from 12 to 84 h) with no arsenite ion was 0.069 ± 0.02 and 0.059 ± 0.04 g/L/h, respectively. At 300 mM As(III), the iron oxidation rate in pure culture (from 12 to 108 h) and coculture (from 12 to 120 h) was 0.044 ± 0.02 and 0.040 ± 0.04 g/L/h, respectively. In the presence of 400 mM As(III), 40.6% and 72.5% of ferrous iron were oxidized in pure and coculture in 168 h. What is more, the oxidation rate of ferrous in pure culture and coculture after 168 h was 22.4% and 42.6% when 500 mM As(III) was added. When the concentration of As(III) reached 600 mM, the ferrous oxidation was totally inhibited. These data indicated that the growth of* L. ferriphilum* YSK was inhibited at higher arsenite concentrations and the strain in coculture showed more resistance to arsenite in contrast with pure culture upon arsenite stress.

The sulfur oxidation in pure and coculture was shown in Figures 2(c)-2(d). In pure culture, the pH value decreased drastically in 48 hours after inoculation. There was no significant variation observed in pH value when the concentration of As(III) was lower than 400 mM, indicating that* At. thiooxidans* A01 had high arsenite resistance. The pure culture was able to decrease the pH down to 1.06 and 1.36 within 168 h at 500 and 800 mM As(III), respectively. It indicated that the growth of* At. thiooxidans* A01 was inhibited. Tolerance to 900 mM was observed in* At. thiooxidans* A01. Compared to the no stress control, the pH value of coculture showed no significant difference up to 500 mM arsenite stress, suggesting that coculture had higher arsenic resistance than pure culture. A slight increase in pH coupled with the increasing arsenite ion concentrations was due to the oxidation of Fe^2+^ to Fe^3+^ that was acid consumption reaction (2Fe^2+^ + 0.5O_2_ + 2H^+^ = 2Fe^3+^ + 2H_2_O). It is interesting that coculture and pure culture with 600 mM As(III) showed similar pH downward trend, since* L. ferriphilum* YSK cannot survive under this condition.

### 3.3. Growth Curve of Pure Culture and Coculture upon As(III) Stress

**Figure 3:**
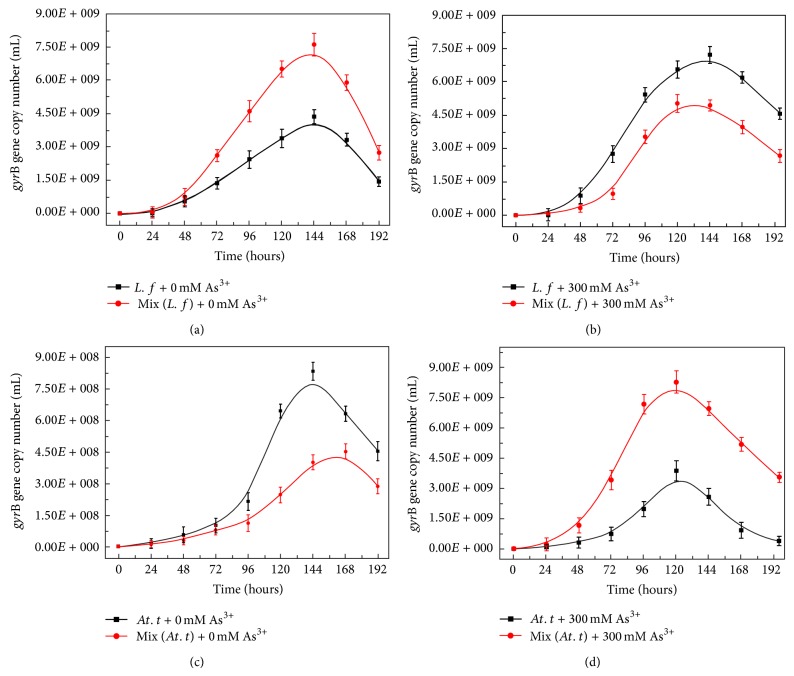
The variation of* gyr*B gene copies of* L. ferriphilum* YSK in pure culture (a) and coculture (b) at 0 (a) and 300 mM As(III) (b); the variation of* gyr*B gene copies of* At. thiooxidans* A01 in pure culture and coculture at 0 (c) and 300 mM As(III) (d). The* error bars* indicate the standard deviation in triplicate.

The growth of* L. ferriphilum* YSK and* At. thiooxidans* A01 in pure culture and coculture with 0 mM and 300 mM As(III) at initial growth was analyzed by real-time PCR. The differences in population dynamics of* L. ferriphilum* YSK and* At. thiooxidans* A01 in pure and coculture were shown in Figure 3.

In coculture* L. ferriphilum* YSK was dominant stain when there was no arsenite stress (Figures 3(a) and 3(c)). The maximum* gyr*B gene copies of* L. ferriphilum* YSK in coculture were about 7.68 ± 0.26 × 10^8^/mL, which was 1.6 times higher than that in pure culture. On the contrary,* At. thiooxidans* A01 was inhibited in coculture with 0 mM As(III), and the maximum* gyr*B gene copies in pure culture and coculture were 8.42 ± 0.34 × 10^9^/mL and 4.55 ± 0.35 × 10^9^/mL, respectively.

In the presence of 300 mM As(III),* At. thiooxidans* A01 became the dominant bacterium in coculture and the growth of* L. ferriphilum* YSK decreased (Figures 3(b) and 3(d)). The* gyr*B gene copies of* At. thiooxidans* A01 in coculture were about 9.8 ± 0.1 × 10^9^/mL, which was 2.45 times higher than that in pure culture at 120 h.* At. thiooxidans* A01 showed higher arsenite resistance which was consistent with the above results. On the contrary, the* gyr*B gene copies of* L. ferriphilum* YSK in coculture were 4.8 ± 0.28 × 10^9^/mL, about 1.5 times lower than that in pure culture at 144 h.

*L. ferriphilum* YSK in coculture grew better than that in pure culture when arsenite was absent. However,* At. thiooxidans* A01 became the dominant bacterium upon arsenite stress. The difference of population dynamics in the presence or absent of As(III) indicated that there were some interactions between iron and sulfur-oxidizing bacterium in coculture.

### 3.4. Response of Pure Culture and Coculture to 300 mM As(III) Shock

**Figure 4:**
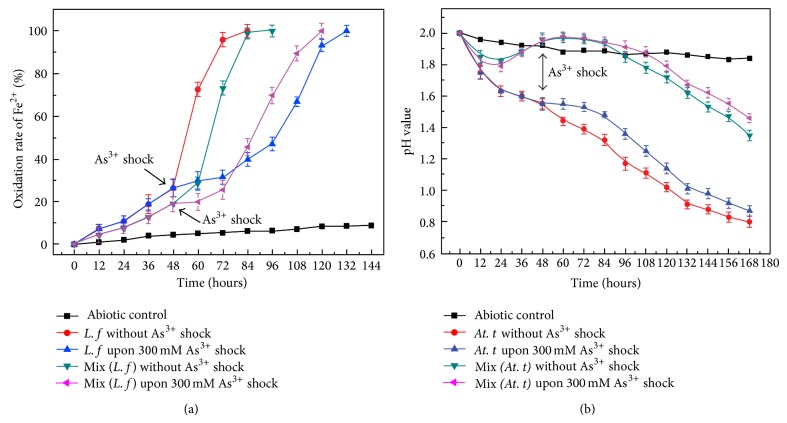
Ferrous iron oxidation rate of* L. ferriphilum* YSK in pure culture and coculture (a) and pH value of* At. thiooxidans* A01 in pure culture and coculture (b) upon 300 mM arsenic shock at 48 h. (The arrow indicates the point of shock.) The* error bars* indicate the standard deviation in replicate samples (*n* = 3).

Cells in pure and coculture at early exponential phase (48 hours) were shocked, respectively, with 300 mM As(III). The bacteria advanced into recession upon arsenite shock and restarted to increase after a short lag time. Ferrous iron was completely oxidized by* L. ferriphilum* YSK in pure culture and coculture in 72 and 96 h without treatment, while 132 h in pure culture and 120 h in coculture with the shock of 300 mM As(III) (Figure 4(a)). The ferrous oxidation was significantly inhibited when shocked by 300 mM As(III) although the pure culture of* L. ferriphilum* YSK got greater influence. The pH value was 0.8 in pure culture and 1.35 in coculture without treatment, while 0.87 in pure culture and 1.46 in coculture with 300 mM As(III) shock (Figure 4(b)). The pH showed no significant change in pure culture and coculture upon 300 mM As(III), confirming that the impact of arsenite on* At. thiooxidans* A01 was less than that on* L. ferriphilum* YSK.

### 3.5. Arsenic-Related Gene Expression by 300 mM As(III) Shock at 48 h

**Figure 5:**
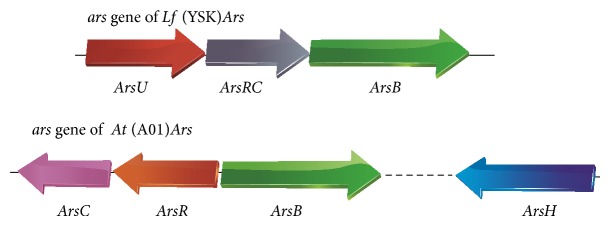
Possible genetic organization of the ORFs in the putative* Lf* (YSK)*Ars* and* At* (A01)*Ars* ars operon based on the finished sequencing and annotation of* L. ferriphilum* YSK and* At. ferrooxidans* A01. The dashed line means the two genes are not connected. The arrow indicates the transcription direction.

In order to study the effect of arsenite shock on the pure culture and coculture at the genetic level, we found out the arsenic resistance genes of* L. ferriphilum* YSK and* At. thiooxidans* A01 by genome sequencing. Each strain contained a set of arsenic gene. The possible genetic organization of the putative* Lf* (YSK)*Ars* and* At* (A01)*Ars* operon was shown in Figure 5. Three arsenic resistance genes of* Lf* (YSK)*Ars*, including* arsU*,* arsRC*, and* arsB*, were all transcribed in the same direction. The* arsU* gene, encoding a protein with unknown function, was located on the upstream of* arsRC*. The* At* (A01)*Ars* gene cluster consisted of* arsC*,* arsR*,* arsB,* and* arsH*. The* arsH* gene was located far away from other genes and was a frequent part of the arsenic resistance system in bacteria.

**Figure 6:**
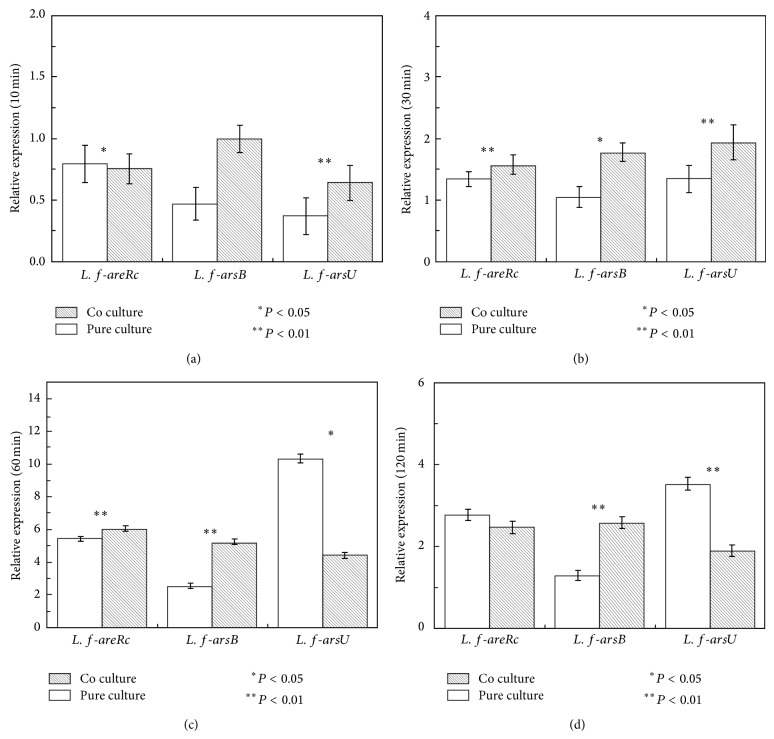
Arsenic resistance gene expression of* L. ferriphilum* YSK in pure culture and coculture upon 300 mM As(III) shock at 10, 30, 60, and 120 minutes determined by real-time PCR: (a) 10 min; (b) 30 min; (c) 60 min; (d) 120 min. The error bars indicate the standard deviation in replicate samples (*n* = 3) and the *P* value indicates the statistical significance between pure and coculture (^∗^ *P* < 0.05; ^∗∗^ *P* < 0.01).

Figure 6 showed the relative expression of arsenite resistance-related genes of* L. ferriphilum* YSK in pure and coculture. Compared to the untreated control,* arsRC*,* arsB,* and* arsU* genes were downregulated at 10 min and upregulated in pure and coculture during 30–120 min. It was worth mentioning that* arsU* gene was upregulated in pure and coculture during 30–120 min, especially at 60 min, indicating that the* arsU* gene was a kind of arsenic resistance gene although its function was not yet clear. At 10 and 120 min, the expression of* arsRC* in pure and coculture showed no significant difference. During 10–120 min, the expression of* arsB* in coculture was continuously upregulated facilitating the pump of arsenite. These data indicated that* L. ferriphilum* YSK in pure and coculture took different strategies upon arsenite shock.

**Figure 7:**
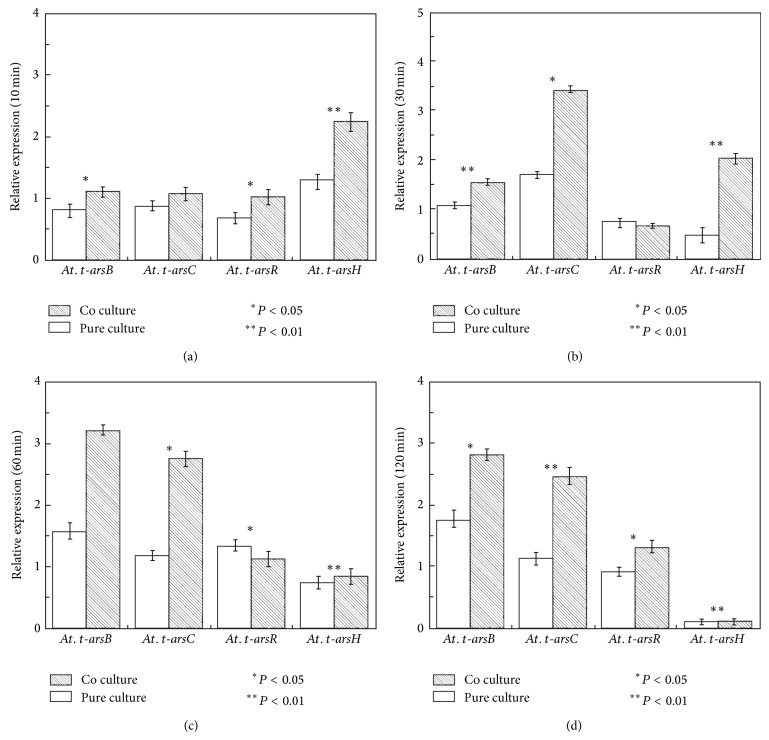
Arsenic resistance gene expression of* At. thiooxidans* A01 in pure culture and coculture upon 300 mM As(III) shock at 10, 30, 60, and 120 minutes determined by real-time PCR: (a) 10 min; (b) 30 min; (c) 60 min; (d) 120 min. The error bars indicate the standard deviation in replicate samples (*n* = 3) and the *P* value indicates the statistical significance between pure and coculture (^∗^ *P* < 0.05; ^∗∗^ *P* < 0.01).

Differential expression of arsenite resistance-related genes of* A. thiooxidans* A01 in pure and coculture was showed in Figure 7. At 10 min,* arsC*,* arsB,* and* arsR* were downregulated in pure and coculture, except that* arsH* was upregulated. The* arsC* and* arsB* genes were upregulated during 30–120 min, and the upregulation of* arsC* and* arsB* gene in coculture was more significant than that in pure culture, confirming that* At. thiooxidans* A01 was more thriving in coculture. The* arsR* gene was downregulated during 10–30 min and upregulated during 60–120 min, and there was no significant difference between pure culture and coculture. It is worth noting that* arsH* gene was significantly upregulated in coculture at 30 min compared to pure culture and then exhibited reduced expression in pure culture and coculture. ArsH protein may play an important role in lessening the toxicity of arsenite.

## 4. Discussion

Arsenic is ubiquitous in the biosphere and is frequently reported to be an environmental pollutant which is toxic to most living cells [33]. In addition, arsenic contamination due to anthropogenic activity (e.g., mining) is increasing in importance in USA, Canada, Australia, Argentina, China, and Mexico [34]. Microorganisms inhabiting metal polluting environments encounter selective pressure to develop metal resistance mechanisms, and many acidophilic microorganisms are resistant to higher concentrations of toxic metals than neutrophilic microorganisms [1].

As important acidophilic leaching microorganisms in the metal-rich environments,* L. ferriphilum* and* At. thiooxidans* were reported as the highly arsenic-resistant strains [5, 35]. In this study, the initial arsenite concentration of* L. ferriphilum* YSK and* At. thiooxidans* A01 was up to 100 mM, while* A. ferrooxidans* BY-3 showed resistance to 60 mM arsenite [5]. Continuous selection could make the bacteria resistant to higher arsenic concentration. The pure culture of* L. ferriphilum *YSK and* At. thiooxidans* A01 could be adopted to 400 mM and 800 mM As(III) after domestication in our study, which was much higher than the strain reported. With the increase of arsenite concentration, the growth capacity and oxidative activity of two strains showed various degrees of inhibition. The results showed that high level of As(III) (≤800 mM) slightly affected S^0^ oxidation of* At. thiooxidans* A01 in pure culture. In addition, the pH showed no significant change in pure culture and coculture upon 300 mM As(III) shock. However, only 40.6% ferrous iron was oxidized in pure culture in 168 h when 400 mM As(III) was added. These data demonstrated that* At. thiooxidans* A01, a member of the highly arsenic-resistant genus [21], showed stronger resistance to arsenite than* L. ferriphilum* YSK. Because the main aim is comparative study of arsenic resistance of acidophilic bacteria in pure culture and coculture, we will research in-depth for arsenic resistance of the single bacteria in the future.

In recent years, a number of researches implied that the metal resistance and bioleaching efficiency in coculture are higher than those in pure culture [36–38]. But the biochemical and genetic mechanisms responsible for metal resistance in acidophilic bacterium remain uncharacterized. The coculture of* L. ferriphilum* YSK and* At. thiooxidans* A01 showed a stronger sulfur and ferrous ion oxidation activity when exposed to arsenite. The pH value of coculture showed no significant difference up to 500 mM arsenite stress compared to the untreated control, indicating that coculture had higher arsenic resistance than pure culture. However, the* gyr*B gene copies of* L. ferriphilum* YSK in coculture were lower than that in pure culture with addition of As(III). We could speculate that* At. thiooxidans* A01 played a determinant role in coculture upon arsenite stress. According the reports, arsenic was partially trapped by sorption and/or coprecipitation, with ferric oxyhydroxy sulfates and oxyhydroxides formed, via the oxidation of dissolved Fe^2+^ in sulfate and iron-rich acidic waters [39]. In addition, Takeuchi et al. indicated that more than half of the arsenic was related to metabolic activity by incorporating into the cytosol fraction, lipid-bound fraction, and the cell surface [40]. It was inferred that* L. ferriphilum* YSK facilitated the growth of* At. thiooxidans* A01 exposed to arsenite stress by iron oxidation reaction and metabolic activity in coculture.

Arsenic detoxification by acidophilic microorganisms is a hot topic. Arsenic efflux system was the most effective way of arsenic detoxification and was encoded by the* ars* operon, which usually has three (*arsRBC*) or five (*arsRDABC*) genes located either in plasmids or chromosomes [4, 21, 41–43]. It was reported that the putative* ars* operon in* L. ferriphilum* ML-04 includes genes similar to* arsRC* and* arsB* [35]. In addition, the arsenic transposon which consisted of* arsR*,* arsC*,* arsD*,* arsA*, and* arsB* was identified in* A. caldus* [21]. They were all described as highly arsenic-resistant strains. In this study, a cluster of arsenic-related genes on the chromosome of* L. ferriphilum* YSK and* At. thiooxidans* A01 were revealed by genome sequencing. The* Lf* (YSK)*Ars* operon includes* arsU*,* arsRC,* and* arsB* while the* At* (A01)*Ars* gene cluster consisted of* arsC*,* arsR*,* arsB,* and* arsH*. The* arsH* gene, contributing to the conversion of arsenate to arsenite, was also found in* Thiobacillus ferrooxidans* and* Yersinia enterocolitica* [19, 44].

In order to study the interaction between* L. ferriphilum* YSK and* At. thiooxidans* A01 at the genetic level, the expression profile of arsenic resistance-related genes in* L. ferriphilum* YSK and* At. thiooxidans* A01 in pure culture and coculture toward 300 mM As(III) shock was analyzed for the first time. Transcriptional analysis showed that they were differentially expressed upon shock, affecting the physiology and growth of bacteria. The expression of* arsC*,* arsB,* and* arsR* under pure culture and coculture was consistently upregulated upon shock after 30 min, lessening the toxicity toward* At. thiooxidans* A01. In addition, the* arsRC* gene in* L. ferriphilum* YSK was downregulated at 10 min and upregulated during 30–120 min in pure culture and coculture. The* arsB* gene in* L. ferriphilum* YSK was downregulated during 10–30 min and upregulated during 30–120 min in pure culture while being upregulated from 10 to 120 min in co culture. Arsenate reductase ArsC which reduced As(V) to As(III) was the most important pathway of arsenate and arsenite detoxification [3]. Butcher and Rawlings reported that* arsRC* genes were induced in* A. ferrooxidans* irrespective of the form of arsenic added [45].* Escherichia coli* could grow in up to 500 mM arsenate after selection, in which gene expressions of* arsC* and* arsB* were significantly increased, and the rate of arsenate reduction was increased 12-fold, while* arsB*, encoding the arsenite membrane pump, resulted in a 4-fold to 6-fold increase in arsenite resistance [46]. What is more, our data revealed that the upregulating of* arsC* and* arsB* in coculture was more significant than that in pure culture, indicating that there is synergetic interaction between* At. thiooxidans* A01* and L. ferriphilum* YSK. It was worth noting that* arsH* gene in* At. thiooxidans* A01, located far away from other genes, showed upregulation at the early stage and reduced at the late stage in pure culture and coculture upon shock. Recent studies place ArsH protein in the family of the NADPH-dependent FMN (flavin mononucleotide) reductases, which might produce hydrogen peroxide, benefiting the conversion of arsenate to arsenite [19, 20, 43]. Some researchers reported that the ArsH protein showed high ferric reductase activity performing an important role for cytosolic ferric iron assimilation* in vivo* [47]. The* arsU* gene had a high expression in the pure culture of* L. ferriphilum* YSK at 60 min, indicating that it was associated with arsenic resistance.

**Figure 8:**
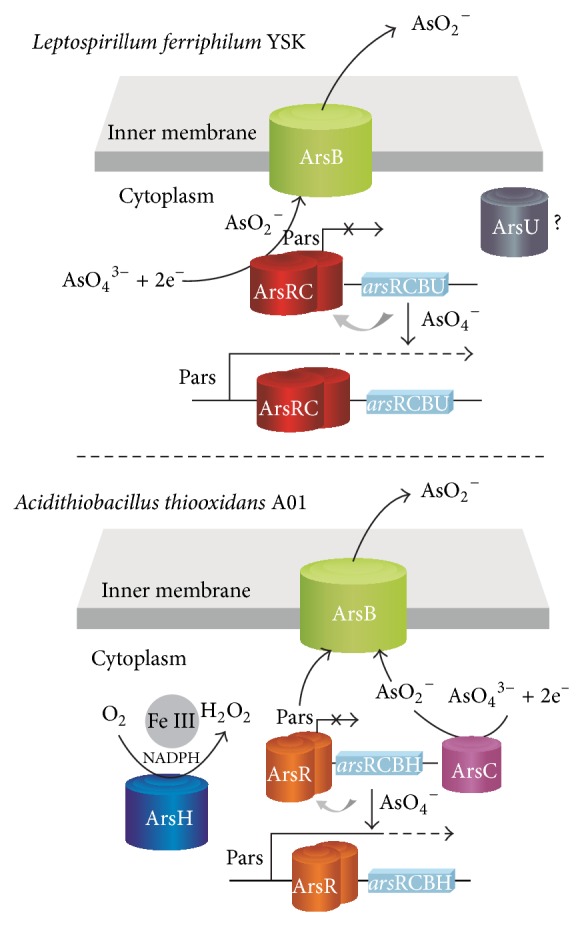
Schematic representation of arsenic interaction with* ars* operon in* L. ferriphilum* YSK and* At. thiooxidans* A01 based on documented models [1, 11, 16, 17, 42, 48–55] and bioinformatics analysis of draft genome sequence. ArsR is an As(III)-responsive transcriptional repressor that binds to the* ars* promoter, repressing transcription. Binding of arsenite to ArsR results in dissociation of the repressor from the DNA and hence gene expression. Arsenate is reduced to arsenite by ArsC. ArsRC also have the function of ArsR and ArsC. Intracellular arsenite is extruded from the cells by ArsB. ArsH is considered to be a H_2_O_2_-forming NADPH-FMN oxidoreductase that might produce hydrogen peroxide which may contribute to the conversion of arsenate to arsenite and played a role in the response to oxidative stress The unknown protein named ArsU was associated with arsenic resistance.

According to the gene expression and draft genome sequence, a model (Figure 8) was proposed to describe the interaction between arsenic and* ars* operons of* L. ferriphilum* YSK and* At. thiooxidans* A01 based on documented models [1, 11, 16, 17, 42, 48–55]. The* Lf* (YSK)Ars gene cluster included* arsU*,* arsRC,* and* arsB*. The ArsRC protein worked as ArsR and ArsC. The* At* (A01)*Ars* gene cluster consisted of* arsC*,* arsR*,* arsB,* and arsH. ArsC located in the plasma membrane functioned to extrude arsenite from the cells and made it accessible to ArsB membrane complex [13, 14, 56]. ArsR, a transacting regulatory protein, controls its own expression and only reacts with the most toxic As(III) [15]. It binds to the* ars* promoter, repressing transcription. Binding of arsenite to ArsR results in dissociation of the repressor from the DNA and hence gene expression. As a H_2_O_2_-forming NADPH:FMN oxidoreductase [57], ArsH might produce hydrogen peroxide involved in conversion of arsenate to arsenite and played a role in the response to oxidative stress caused by As(III). ArsU, a hypothetical protein, may be associated with arsenic resistance. Further study is needed for the function of ArsU and ArsH in* L. ferriphilum* YSK and* At. thiooxidans* A01.

## 5. Conclusion

In this study, a coculture system including* L. ferriphilum* YSK and* At. thiooxidans* A01 was constructed. The As(III) resistance of* L. ferriphilum* YSK and* At. thiooxidans* A01 in pure culture and coculture were investigated by physiological and transcriptional analysis. The coculture showed a stronger metabolic activity, indicating that there is a synergetic interaction between* At. thiooxidans* A01 and* L. ferriphilum* YSK.* At. thiooxidans* A01, which could adapt up to 800 mM As(III) after adaptation, showed higher arsenic resistance in pure culture and coculture than* L. ferriphilum* YSK. What is more,* At. thiooxidans* A01 was dominant in coculture and the cell number was 2.45 times higher than that in pure culture at 300 mM As(III). Transcriptional analysis revealed that the arsenic efflux system in the coculture was more active than that in pure culture. Arsenic efflux system was the important pathway of arsenite detoxification in* L. ferriphilum* YSK and* At. thiooxidans* A01.* arsH* and* arsU* showed differential expression toward arsenite although their roles need further research. Coculture of* L. ferriphilum* YSK and* At. thiooxidans* A01 provided insights into microbial interactions in acidic environment, which will facilitate its effective application in the bioleaching system.

## Figures and Tables

**Table 1:** Specific primers used for general and real-time PCR.

Primer	Primer sequences (5′-3′)	Temperature (°C)	Amplicon length (bp)	qPCR quantification target gene and function
Lf(YSK)-F Lf(YSK)-R	GAAAACACTTGAGGACGG CGGATAAAACGGTTGATT	56 56	168	*gyr*B gene of *L*. *ferriphilum* YSK

At(A01)-F At(A01)-R	GACCCGTACCCTCAATCA CGGTTTCACTTCACTGGA	58 58	175	*gyr*B gene of *At*. *thiooxidans* A01

Lf-ArsB-F Lf-ArsB-R	GGAGGCAAGGTCTGCGAGAA GGGAAAAGGGCGGCTCATTC	62 65	138	*arsB* gene of *L*. *ferriphilum* YSK, membrane-associated arsenite export pump

Lf-ArsU-F Lf-ArsU-R	GCCAATCCGGTCCTGGTTCA CCCATTTTCTTTTCGGGCTTTC	65 64	146	*arsU* gene of *L*. *ferriphilum* YSK, arsenic metabolism related protein of unknown function

Lf-ArsRC-F Lf-ArsRC-R	GAACGACAGGCATTTTGAGT GACGAGGACCTTTCTGACCA	55 57	138	*arsRC* gene of *L*. *ferriphilum* YSK, arsenate reductase/phosphotyrosine protein phosphatase gene

At-ArsB-F At-ArsB-R	AAAAGTCGCTGTGGGTGAAA GTTGGGTGCTTGTATTGCTG	58 56	112	*arsB* gene of *At*. *thiooxidans* A01, arsenical pump membrane protein

At-ArsC-F At-ArsC-R	ATGGCGGGTCCGAGGTAGGC GAGGGGTTTGCCACGGAAGG	67 66	128	*arsC* gene of *At*. *thiooxidans* A01, arsenate reductase

At-ArsH-F At-ArsH-R	TCCGACCATTCTACCAGTTCC AGCGGCTATTACGCCATTTT	58 59	127	*arsH* gene of *At*. *thiooxidans* A01, arsenical resistance protein

At-ArsR-F At-ArsR-R	ACTAATGCCCGAACTACGCT TATTGCCGAACATCTGGACC	58 56	141	*arsR* gene of *At*. *thiooxidans* A01, transcriptional regulator, a negative regulator of the *ars* operon
